# Possible proteomic biomarkers for the detection of pancreatic cancer in oral fluids

**DOI:** 10.1038/s41598-020-78922-x

**Published:** 2020-12-15

**Authors:** O. Deutsch, Y. Haviv, G. Krief, N. Keshet, R. Westreich, S. M. Stemmer, B. Zaks, S. P. Navat, R. Yanko, O. Lahav, D. J. Aframian, A. Palmon

**Affiliations:** 1 Institute of Dental Sciences, Faculty of Dental Medicine, The Hebrew University of Jerusalem, Jerusalem, Israel; 2 Salivary Gland Clinic and Saliva Diagnostic Laboratory, Department of Oral Medicine, Sedation and Maxillofacial Radiology, Sjogren’s Syndrome Center, Hadassah Medical Center, The Hebrew University of Jerusalem, Jerusalem, Israel; 3 Department of Internal Medicine B, Soroka Medical Center, Beer-Sheva, Israel; 4 Faculty of Health Sciences, Ben-Gurion University of the Negev, Beer-Sheva, Israel; 5 Rabin Medical Center, Davidoff Center, Petach Tiqwa, Affiliated to the Sackler Faculty of Medicine, Tel Aviv University, Tel Aviv, Israel; 6 Department of Oral Medicine, Sedation and Maxillofacial Imaging, Faculty of Dental Medicine, Hebrew University – Hadassah, Jerusalem, Israel

**Keywords:** Biochemistry, Biological techniques, Biotechnology, Cancer, Computational biology and bioinformatics, Molecular biology, Biomarkers, Diseases, Medical research, Molecular medicine, Oncology

## Abstract

The 80% mortality rate of pancreatic-cancer (PC) makes early diagnosis a challenge. Oral fluids (OF) may be considered the ultimate body fluid for non-invasive examinations. We have developed techniques to improve visualization of minor OF proteins thereby overcoming major barriers to using OF as a diagnostic fluid. The aim of this study was to establish a short discriminative panel of OF biomarkers for the detection of PC. Unstimulated OF were collected from PC patients and controls (n = 30). High-abundance-proteins were depleted and the remaining proteins were analyzed by two-dimensional-gel-electrophoresis and quantitative dimethylation-liquid-chromatography-tandem mass-spectrometry. Label-free quantitative-mass-spectrometry analysis (qMS) was performed on 20 individual samples (n = 20). More than 100 biomarker candidates were identified in OF samples, and 21 had a highly differential expression profile. qMS analysis yielded a ROC-plot AUC value of 0.91 with 90.0% sensitivity and specificity for a combination of five biomarker candidates. We found a combination of five biomarkers for PC. Most of these proteins are known to be related to PC or other gastric cancers, but have never been detected in OF. This study demonstrates the importance of novel OF depletion methodologies for increased protein visibility and highlights the clinical applicability of OF as a diagnostic fluid.

## Introduction

Pancreatic cancer (PC) often remains undetected until the late stages of the disease. Each year approximately 37,000 Americans are diagnosed with PC, furthermore, 33,000 Americans and more than 42,000 Europeans die from pancreatic cancer annually^1^. PC was the 4th leading caner type for estimated deaths in the USA in 2012 and 2013^2–4^.

The median survival time for PC is nine to 12 months with an overall 5-year survival rate of 3%. The high mortality rate is due in part to the fact more than 50% of patients with PC have metastatic disease at the time of diagnosis. The 50% recurrence of PC following surgical removal, suggests that PC is relatively refractory to current treatments.

No specific tumor marker for the diagnosis of PC has been identified, complicating early diagnosis. Therefore, extensive genomic, transcriptomic, and proteomic studies are being performed to identify candidate markers by employing high-throughput systems capable of large cohort screening. Currently, early detection of pancreatic cancer in high-risk patients is done using highly invasive means (Endoscopic ultrasound combined with fine-needle-aspiration). These methods cause discomfort, require an expert team and are very expensive, making them useless as screening tools. The lack of a single diagnostic marker suggests that only a combination of biomarkers will be able to provide the appropriate combination of high sensitivity and specificity. Biomarker discovery using novel technologies can improve prognostic upgrading and pinpoint new molecular targets for innovative therapy.

Over the last decade OF have been recognized as a "diagnostic window to the body"^5^. This is due to the fact that despite the apparent low degree of overlap between OF and plasma, the distribution found across Gene Ontological categories, such as molecular function, biological processes, and cellular components, is very similar^6^.

Many centers, including our department, have taken advantage of the non-invasive access to this readily available body fluid. Furthermore the composition of OF is known and therefore fluctuations can be used to monitor diseases and physiological changes^7^. The positive aspects of OF compared to serum as a diagnostic fluid for practitioners include simple collection (of adequate volumes), storage and shipment. Procurement is also safer than venipuncture, limiting exposure to infectious agents. The non-invasive, painless collection reduces fear and enhances compliance when repeated samples are needed over time. The non-clotting nature of the fluid makes it ideal for diagnostic purposes^8^.

Analysis of OF using proteomics has been hindered by the presence of high abundant proteins such as salivary alpha amylases (sAA)^9–12^ albumins (alb)^13^ and immunoglobulins (Ig)^12,13^ which conceal or reduce the separation sensitivity of other proteins.

There are two main advantages to high abundant protein depletion followed by 2DE: (i) gel resolution is increased because the levels of the low abundant proteins in the proteomic map are relatively higher and (ii) important low abundant proteins are revealed when the overlapping high abundant protein spots are removed. Low abundant proteins can also be exposed by using qMS.

We have developed and successfully used techniques to remove the high abundant proteins in OF, thereby improving protein visualization^14–16^. We hypothesize that OF composition will be altered by pancreatic cancer. The similarity of the structure of the salivary and pancreatic glands may cause the salivary glands to function as a biological amplifier and to produce proteins in response to PC which will be detectable in OF. This phenomenon has been reported in breast cancer patients, (the structure of the mammary glands is also similar to the salivary glands). C-erb-b2 a breast cancer marker was produced by the salivary glands and detected in the saliva of breast cancer patients^17^.

The aim of this study was to identify and develop an early detection assay for PC based on OF, to characterize OF proteins following removal of the high abundance proteins, and to identify candidate biomarkers for PC.

## Materials and methods

### Ethical approval

The OF accumulation protocol was approved by the Ethical Committee, Rabin Medical Center, Beilinson Hospital, Request No. 0053-09-RMC. Informed consent was obtained according to the instructions of the Ethical Committee. All procedures were carried out in accordance with relevant guidelines and regulations.

### OF collection, patients and healthy volunteers

Unstimulated OF flow was collected for 5 min using the spitting method^18^ into pre-calibrated tubes. All participants refrained from eating, drinking and brushing their teeth 1 h prior to saliva collection. Patients did not take their medications, including sialagogues, before saliva collection.

Volunteers rested for 10 min before saliva collection, sitting in an upright position and in a quiet room and were asked not to speak or leave the room until after the saliva was collected. Saliva samples were immediately placed on ice and then centrifuged at 14,000 g for 20 min at 4 °C to remove insoluble materials, cell debris and food remnants. The supernatant of each sample was collected and protein concentration was determined using the Bio-Rad Bradford protein assay (Bio-Rad, Hercules, CA, USA) as previously described^19^.

OF were collected from 31 males; 15 PC patients and 16 healthy, age matched controls. Controls did not take any medications known to cause xerostomia (supplementary data A), had no complaints of oral dryness and no evidence oral mucosal diseases was detected following examination. 2 patients in the PC group were undergoing chemotherapy at the time of collection and were therefore excluded from the OF pool. Salivary flow rate was calculated. OF samples were divided into two groups: (1) for to 2DE and Demethylation MS analysis (described below), samples were pooled according to the amount of total protein in each individual sample. 2) For label-free qMS, individual samples were used.

### sAA affinity removal

Amylase was removed from the pooled OF using an amylase removing device. 600 µL of water was hand pressed (20 s) through the device to moisturize the substrate. Thereafter, 1 mL of pooled OF (in two aliquots of 500 µL) was hand pressed and filtered (120 s) through the amylase removing device. The resultant 1 mL of filtrated OF was amylase-free, as previously described^14^.

### Alb and IgGs removal, capturing and elution

In order to remove alb and IgGs the ProteoPrep Immunoaffinity alb and IgG Depletion Kit (Sigma-Aldrich, St Louis, MO, USA) were used as previously described^15^ Protein concentration was measured again as before, using the Bio-Rad Bradford protein assay (Bio-Rad, Hercules, CA, USA)^19^.

The triple depleted OF were divided to 2 tubes for 2DE and quantitative MS analysis and frozen at − 80 °C and lyophilized overnight. Sediments (products (deposits) of lyophilization processes) for 2DE were dissolved in 7M urea, 2M thiourea and 4% 3-[(3-cholamidopropyl) dimethylammonio]-1-propane-sulfonate (CHAPS) and stored at − 20 °C until analysis.

### Two-dimensional sodium dodecyl sulfate polyacrylamide gel electrophoresis (2DE)

For analytical gels, 100 µg of protein were rehydrated then subjected to isoelectrofocusing in 18 cm long second dimension gels, pH 3–10 NL as previously described^20^. To prepare the gel strips for separation in the second dimension they were soaked twice for 15 min in an SDS-PAGE equilibration buffer as previously described^14^. For the second dimension, strips were embedded in 0.5% w/v agarose containing a trace of bromophenol blue and loaded onto hinged spacer plates (20 cm × 20.5 cm; Bio-Rad, Hercules, CA, USA) using 9.5–16.5% SDS polyacrylamide gradient gel electrophoresis. The same running and staining apparatus at a constant current of 30 mA per gel at 10 °C was used for all samples. Gels were silver stained with SilverQuest kit (Invitrogen, Carlsbad, CA, USA).

### Imaging and statistical analysis

Gels were scanned using a computer GS-800 calibrated densitometer (Bio-Rad, Hercules, CA, USA) and spots were detected and quantified using PDQuest software V 6.2.0 (Bio-Rad, Hercules, CA, USA). In order to overcome several of the known limitations of 2D gel analysis that occur as a result of gel to gel variation, and also variability in staining^14^, all samples were run simultaneously for the first and second dimensions. Normalization with PDQuest was performed using the total density in image method to semi-quantify spot intensities and to minimize staining variation between gels^14^.

### 2DE Mass-spectrometry (MS) identification

For MS identification, a 2DE containing 100 µg of protein was prepared and fixed in 50% (v/v) ethanol, 12% (v/v) acetic acid for 2 h. Proteins were visualized by staining with a SilverQuest staining kit for MS compatible silver staining (SilverQuest, Invitrogen, Carlsbad, CA, USA). Electrophoretically separated spots were excised from the gels, and in-gel reduced (10 mM Dith-9 iothreitol, incubated at 6 °C for 30 min), alkylated (10 mM iodoacetamide, at room temperature for 30 min) and proteolyzed with trypsin (overnight at 37 °C using modified trypsin, Promega at a 1:100 enzyme-to-substrate ratio). The resulting tryptic peptides were resolved by reversed-phase chromatography on 0.1·200-mm fused silica capillaries (J&W, 100 µm ID) packed with Everest reversed phase material (Grace Vydac, CA, USA). The peptides were eluted with a 45 min gradient of 5 to 95% (v/v) of acetonitrile with 0.1% (v/v) formic acid in water at flow rates of 0.4 ll min. Mass spectrometry was performed by an ion-trap MS (Orbitrap; Thermo) in a positive mode using a repetitively full MS scan followed by collision induced dissociation (CID) of the five most dominant ions selected from the first MS scan. The MS data were clustered and analyzed using Sequest software (version 3.31; J. Eng and J. Yates, University of Washington and Finnegan, San Jose, USA) and Pep-Miner^21^ searching against the human part of the Uniprot database (2014_03, https://www.uniprot.org/). The results were filtered according to the Xcorr value (1.5 for singly charged peptides, 2.2 for doubly charged peptides and 3 for triply charged peptides).

### Quantitative mass-spectrometry (MS)

#### Protein extraction and proteolysis

The proteins in 8M Urea were reduced with 2.8 mM DTT (60 °C for 30 min), modified with 8.8 mM iodoacetamide in 100 mM ammonium bicarbonate (room temperature for 30 min) and digested in 2M Urea, 25 mM ammonium bicarbonate with modified trypsin (Promega) at a 1:50 enzyme-to-substrate ratio, overnight at 37 °C. In order to achieve full cleavage, a second 4 h digestion was performed at 37 °C.

#### Demethylation MS analysis

As described previously by Krief et al.^7^, the resulting peptides were desalted using C18 Stage tips, dried and re-suspended in 50 mM Hepes (pH 6.4). Labeling by Dimethylation was done in the presence of 100 mM NaCBH_3_ (Sterogene cat#9704 1M), by adding Light Formaldehyde (35% Frutarom cat#5551810, 12.3M ) to the pooled control sample, and Heavy Formaldehyde (20% w/w, Cambridge Isotope laboratories cat#CDLM-4599-16.5M) to the pooled PC sample to a final concentration of 200 mM. Following 1 h of incubation at room temperature the pH was raised to 8 and the reaction was incubated for another hour at room temperature. Neutralization was done with 25 mM ammonium bicarbonate for 30 min, and equal amounts of the light and heavy peptides were mixed, cleaned on a C18 stage tip, dried and re-suspended in 0.1% formic acid.

Peptides were resolved by reverse-phase chromatography on 0.075 × 200-mm fused silica capillaries (J&W) packed with Reprosil reverse phase material (Dr. Maisch GmbH, Germany). The peptides were eluted with linear 215 min gradients of 7 to 40% and then for 8 min at 95% acetonitrile with 0.1% formic acid in water at flow rates of 0.25 μl/min. Mass spectrometry was performed using an ion-trap mass spectrometer (Orbitrap, Thermo) in a positive mode using a repetitively full MS scan followed by collision induced dissociation (CID) of the 7 most dominant ions selected from the first MS scan.

The MS data was analyzed using Sequest 3.31 software (J. Eng and J. Yates, University of Washington and Finnegan, San Jose) searching the human part of the NCBI-NR database. Quantitation was performed using the PepQuant algorithm of Bioworks and "in house" software.

#### Label free MS analysis

20 individual samples (from 10 PC patients and 10 healthy volunteers) were analyzed using Label free analysis following the depletion of high abundance proteins. The tryptic peptides were desalted using C18 tips, dried and re-suspended in 0.1% formic acid. The peptides were resolved by reverse-phase chromatography on 0.075 × 200-mm fused silica capillaries (J&W) packed with Reprosil reversed phase material (Dr Maisch GmbH, Germany). The peptides were eluted as described above. A wash run and one blank injection were performed between the samples to make sure there was no cross contamination^7^.

The MS data was analyzed using MaxQuant 1.2.2.5 software (Mathias Mann's group) searching against the human section of the Uniprot database and quantified by label free analysis using the same software. Statistical analysis was done using Perseus software (Mathias Mann's group).

#### Bio-statistical analysis

Dr. Yoav Smith (Head of the Genomic Data Analysis Unit, The Hebrew University, Jerusalem) was our consultant for the analysis. Briefly, label-free qMS results were initially analyzed utilizing Matlab software R2013a (The MathWorks, Inc. USA). Data was then presented in a Volcano plot using the vertical axis for the p-values and the horizontal axis for the log 2 ratio values. By using a threshold of less than 0.05 for the p-values, and a fold change of + or − 2 for the absolute log 2 ratios, proteins with the largest statistically significant expression change were chosen. Furthermore, for the combined protein group the predicted probability for each subject was obtained and was used to construct receiver operating characteristic (ROC) curves. The standard error of the area under the curve (AUC) value and the 95% confidence interval (CI) for the ROC curve were computed as previously described^22^. The sensitivity and specificity for the combined biomarkers were estimated by identifying the cutoff-point of the predicted probability that yielded the highest sum of sensitivity and specificity.

## Results

The mean age of the 15 PC patients was 65.7 ± 13.24 years, and the mean age of the 16 healthy age-matched controls was 56.5 ± 3.3 years. The average time from PC diagnosis to OF collection was ~ 7 months.

72% of the patients were diagnosed with stage IV and the rest with stage III. All the PC patients took medications regularly, and their tendency to cause xerostomia was checked (supplementary data A), only 2 patients used medicines known to cause dry mouth in more than 10% of individuals.

The study was divided into sections: 1. Proteomic analysis on pooled samples using 2DE and dimethylation-qMS. 2. Analysis of individual samples using label-free qMS.

### Dimethylation MS analysis of pooled PC and control samples

Dimethylation followed by LC–MS/MS of PC and control OF samples exposed 182 proteins (supplementary data B). 21 proteins showed an extended differential profile with a 3 to 50-fold change in expression. 37 proteins had a 2 to threefold expression change (see Table 1 for details). Table 1A refers to publications implicating 19 of our 21 identified proteins as biomarker candidates for PC or other cancers. None of these proteins has ever been detected in OF of PC patients.

**Table 1:** Proteins identified by Dimethylation MS analysis of pooled PC and control samples. A. Highly differentiated expression profile (above threefold). B 2 to threefold expression profile differences.

									Previously identified	
Serial no	Protein identification	Accession no	MW (Da)	Matched peptides	Average Ratio PC/C	Sample origin	Not Identified	Not Identified	PC biomarkers	Other cancer biomarkers
**A**										
1	Histone H4	P62805	11,360	3	0.02	HNSCC*, Saliva				[43, 44]
2	Histone H2B type 1-B	P33778	13,942	2	0.03	Pancreatic tumor tissue			[45]	
3	6-phosphogluconate dehydrogenase, decarboxylating	P52209	53,106	2	0.04	FNA of PTC**				[46]
4	Basic salivary proline-rich protein 2 precursor	P02812	40,775	2	0.05	Saliva				[44]
5	Histone H2B type 1-A	Q96A08	14,159	2	0.06	HNSCC*				[43]
6	Azurocidin precursor	P20160	26,869	3	0.07		x	x		
7	Apolipoprotein A-I precursor	P02647	30,759	7	0.08	Biological sample				[27]
8	Alpha-amylase 1 precursor	P04745	57,731	31	0.16	Saliva				[44]
9	Myeloperoxidase precursor	P05164	83,815	9	0.16	Blood			[28]	
10	Protein S100-A8	P05109	10,828	6	0.19	Human Pancreatic Cell-line			[30]	
11	Transthyretin precursor	P02766	15,877	6	0.22	Serum			[29]	
12	Lipocalin-1 precursor	P31025	19,238	12	0.23	Human Pancreatic Cell-line			[30]	
13	Protein S100-A9	P06702	13,234	7	0.24	Biological sample, Saliva			[47]	[44]
14	Short palate, lung and nasal epithelium carcinoma-associated protein 2 precursor	Q96DR5	26,995	6	0.24	Saliva				[48]
15	Hemoglobin subunit alpha	P69905	15,248	9	0.25	Pancreatic tumor tissue,Saliva			[45]	[44]
16	Small proline-rich protein 2A	P35326	7960	3	0.25		x	x		
17	Hemoglobin subunit delta	P02042	16,045	2	0.26	Tissue and Serum				[49]
18	Transketolase	P29401	67,835	11	3.18	pancreatic ductal tissue			[31]	
19	Keratin, type I cytoskeletal 10	P13645	59,475	2	4.57	Pancreatic cancer tissue			[50]	
20	Hemopexin precursor	P02790	51,643	13	4.99	Plasma, Saliva			[32]	[44]
21	Alpha-2-macroglobulin precursor	P01023	163,174	41	8.06	Plasma, Saliva				[44,51]

Serial No	Protein Identification	Accession No	MW (Da)	Peptides No	avg Ratio (Patient/ Healthy)					
**B**										
1	Fibrinogen alpha chain precursor	P02671	94,914	5	0.31					
2	Serum albumin precursor	P02768	69,322	14	0.31					
3	Hemoglobin subunit beta	P68871	15,988	20	0.33					
4	Vitamin D-binding protein precursor	P02774	52,929	5	0.34					
5	Complement C3 precursor	P01024	187,029	27	0.35					
6	Alpha-1B-glycoprotein precursor	P04217	54,239	5	0.35					
7	Alpha-1-acid glycoprotein 1 precursor	P02763	23,497	8	0.37					
8	Actin, cytoplasmic 1	P60709	41,710	12	0.38					
9	L-lactate dehydrogenase B chain	P07195	36,615	2	0.39					
10	Leukotriene A-4 hydrolase	P09960	69,241	2	0.40					
11	Fibrinogen gamma chain precursor	P02679	51,479	6	0.40					
12	Involucrin	P07476	68,427	2	0.44					
13	Metalloproteinase inhibitor 1 precursor	P01033	23,156	2	0.46					
14	Glyceraldehyde-3-phosphate dehydrogenase	P04406	36,030	8	0.48					
15	Fibrinogen beta chain precursor	P02675	55,892	5	0.49					
16	Protein-glutamine gamma-glutamyltransferase E precursor	Q08188	76,584	6	0.49					
17	Beta-2-glycoprotein 1 precursor	P02749	38,273	3	0.49					
18	Keratin, type I cytoskeletal 13	P13646	49,555	3	0.49					
19	Ig alpha-1 chain C region	P01876	37,631	27	0.54					
20	Serotransferrin precursor	P02787	77,000	56	0.54					
21	Vimentin	P08670	53,619	5	0.54					
22	Alpha-1-acid glycoprotein 2 precursor	P19652	23,588	2	0.54					
23	Desmoglein-1 precursor	Q02413	113,644	3	0.57					
24	Ig kappa chain C region	P01834	11,602	14	0.58					
25	Zinc-alpha-2-glycoprotein precursor	P25311	33,851	32	0.58					
26	Cornulin	Q9UBG3	53,502	4	0.58					
27	Phosphoglycerate mutase 1	P18669	28,786	2	0.58					
28	Ig gamma-1 chain C region	P01857	36,083	11	0.58					
29	Ig heavy chain V-III region VH26 precursor	P01764	12,574	6	0.58					
30	Complement factor B precursor	P00751	85,479	2	0.59					
31	Aldehyde dehydrogenase, dimeric NADP-preferring	P30838	50,347	3	0.60					
32	Cystatin-S precursor	P01036	16,204	10	1.91					
33	Ig kappa chain V-III region SIE	P01620	11,768	6	1.94					
34	Keratin, type II cytoskeletal 1	P04264	65,978	4	2.15					
35	Keratin, type II cytoskeletal 2 epidermal	P35908	65,825	4	2.29					
36	Alpha-actinin-1	P12814	102,993	4	2.49					
37	Prolactin-inducible protein precursor	P12273	16,562	10	2.59					

*NSCC—Human Head-and-Neck Squamous Cell Carcinomas tissue; * FNA of PTC—Fine Needle Aspiration of Papillary Thyroid Cancer.

### 2DE and MS analysis of pooled PC and control samples

**Figure 1:**
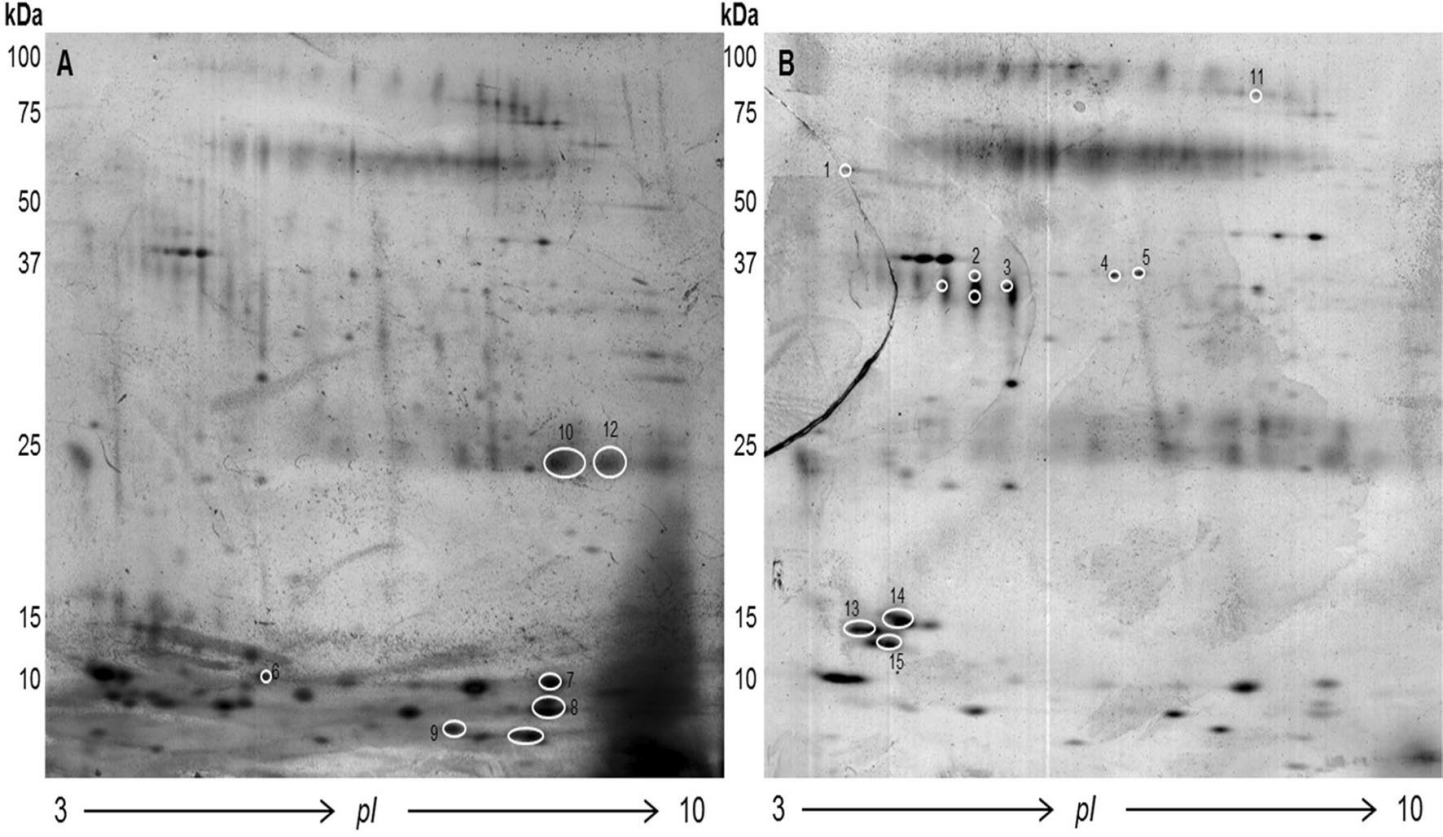
Silver-stained 2DE gels of pooled oral fluid samples after triple depletion (100 µg). (**A**) Control group and (**B**) PC group. Numbered spots were found with an OD change above fivefold (PDQuest software, Bio-Rad, USA) and identified by MS.

2DE of pooled triple-depleted OF samples from healthy controls (Fig. 1A) and PC patients (Fig. 1B) was performed. PDQuest analysis revealed 360 protein spots, and 72 had an expression change of more than threefold. 15 spots with expression changes greater than fivefold were chosen for MS analysis. Only spots identified in both maps were further analyzed by MS (supplementary data C). Of the twenty proteins identified, 12 were newly identified; Ig kappa chain V-I region AG (P01593), Ig kappa chain V–I region DEE (P01597), Polymeric immunoglobulin receptor P01833, Ig alpha-1 chain C region (P01876), Cystatin-B (P04080), Protein disulfide-isomerase (P07237), Leukocyte elastase inhibitor (P30740), Beta-2-microglobulin (P61769), Fatty acid-binding protein, epidermal (Q01469), Serpin(Q9UIV8), Tumor necrosis factor ligand superfamily member 13B (Q9Y275), IgGFc-binding protein (Q9Y6R7). Of the 8 proteins also found in the qMS results, 5 had a similar trend; Ig kappa chain C region (P01834), Ig mu chain C region (P01871), Serum albumin (P02768), Leukotriene A-4 hydrolase (P09960), Hemoglobin subunit beta (P68871). The remaining *3 showed an opposite trend*; Ig kappa chain V-III region SIE (P01620), Zinc-alpha-2-glycoprotein (P25311), Hemoglobin subunit beta (P68871) and Lipocalin-1 (P31025).

### Label free qMS on individual samples

**Figure 2:**
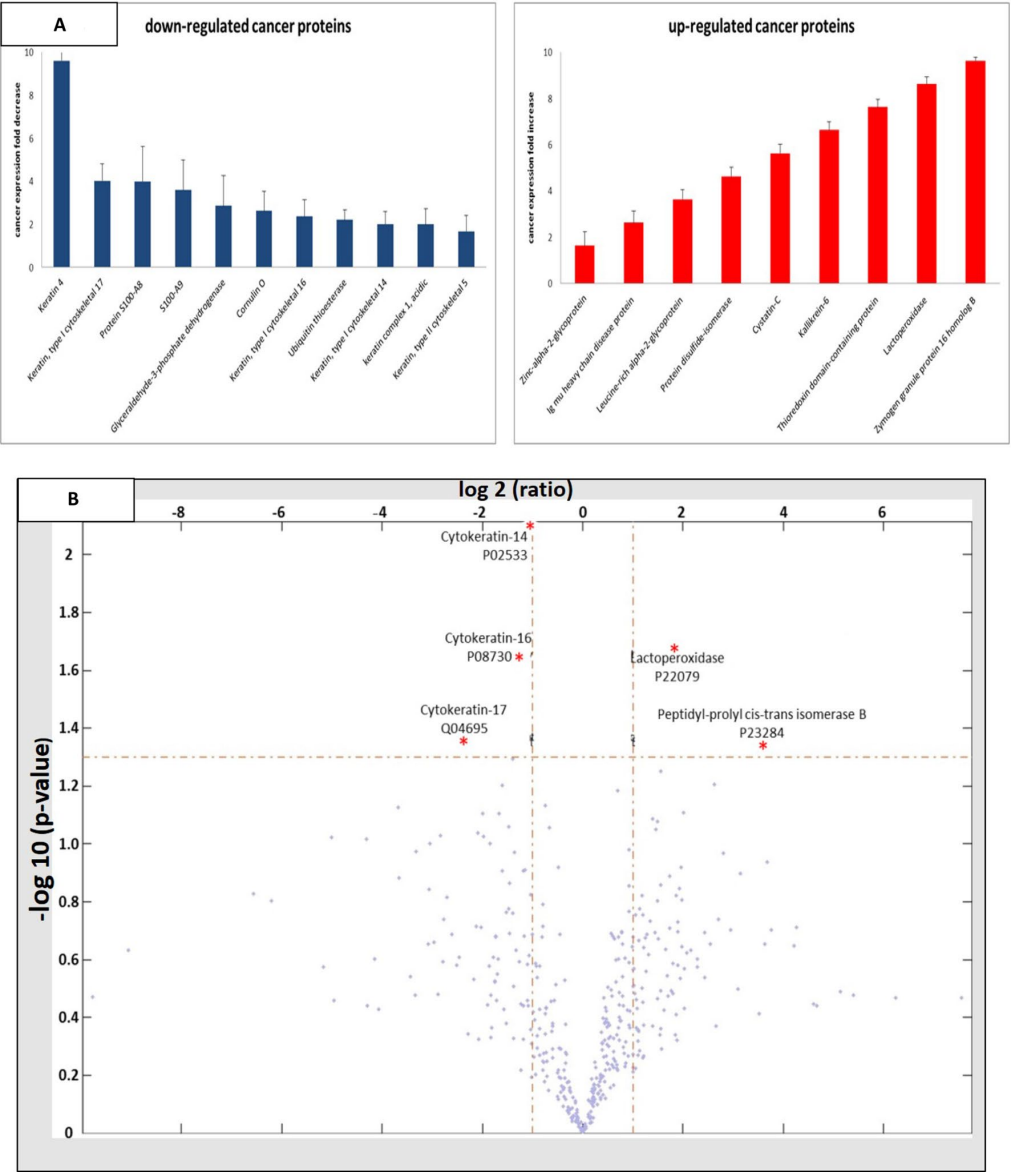
(**A**) Graphical illustrations of 20 proteins with significantly increased expression (*p* < 0.05) after normalization, found in at least 6 subjects per group. (**B**). Volcano plot. Red asterisks represent five proteins with the largest statistically significant changes in expression. (**C**). ROC curve utilizing five biomarkers (P02533, P22079, P08730, Q04695 and P23284) yielded an AUC value of 0.910, with 90.0% sensitivity and 90.0% specificity.

This extensive examination led to the identification of 480 proteins. MS results show the relative expression profile of the proteins in each sample. An average expression ratio was calculated for each protein. 71 proteins were down regulated by more than twofold in PC samples, among them 34 by more than threefold. 92 proteins were up regulated by more than twofold, out of them 46 by more than threefold. The subsequent statistical analysis (t test, *p* value < 0.05), showed 39 proteins with an average change in expression profile of more than twofold. The proteins were grouped according to the number of subjects in which they were found; less than 6 subjects and more than 6 subjects. For example, S100-A9 was found in OF samples of all subjects, and decreased significantly (*p* < 0.05) by more than threefold in PC patients [Table 2, Fig. 2A].

**Table 2:** Individual sample analysis (n = 20) by label free qMS. A. Proteins identified in at least 6 control and PC subjects with an average differential expression (*P* < 0.05). B. Proteins with an average differential expression (*P* < 0.05), with no minimum number of subjects.

Serial no	Protein description	Accesion no	MW (kDa)	PC/H	*P* value	No. of PC samples analysed	No. of Healthy samples analysed
**A**							
1	Keratin 4	P19013	63.91	0.10	0.04	6	8
2	Keratin, type I cytoskeletal 17	Q04695	48.11	0.25	0.03	8	10
3	Protein S100-A8	P05109	10.83	0.25	0.04	10	10
4	S100-A9	P06702	13.24	0.28	0.05	10	10
5	Glyceraldehyde-3-phosphate dehydrogenase	P04406	36.05	0.35	0.05	9	10
6	Cornulin O	Q9UBG3	53.53	0.38	0.02	9	9
7	Keratin, type I cytoskeletal 16	P08779	51.27	0.42	0.01	10	10
8	Ubiquitin thioesterase	Q9UGI0	80.97	0.45	0.02	7	7
9	Keratin, type I cytoskeletal 14	P02533	51.62	0.50	0.00	10	10
10	keratin complex 1, acidic	A2A5Y0	47.12	0.50	0.05	5	7
11	Keratin, type II cytoskeletal 5	P13647	62.38	0.60	0.04	10	10
12	Zinc-alpha-2-glycoprotein	P25311	34.26	1.63	0.03	10	10
13	Ig mu heavy chain disease protein	P04220	43.06	2.63	0.03	6	7
14	Leucine-rich alpha-2-glycoprotein	P02750	38.18	3.63	0.05	10	9
15	Protein disulfide-isomerase	P07237	57.12	4.63	0.04	8	9
16	Cystatin-C	P01034	15.8	5.63	0.05	10	9
17	Kallikrein-6	Q92876	26.86	6.63	0.03	8	8
18	Thioredoxin domain-containing protein	Q9BRA2	13.94	7.63	0.02	8	8
19	Lactoperoxidase	P22079	80.29	8.63	0.01	10	9
20	Zymogen granule protein 16 homolog B	Q96DA0	22.74	9.63	0.03	10	10
**B**							
1	Beta-actin-like protein 2	Q562R1	42	0.10	0.04	2	4
2	Apolipoprotein A-I	P02647	30.78	0.15	0.05	4	6
3	Purine nucleoside phosphorylase	P00491	32.12	0.20	0.05	5	4
4	Annexin A1	P04083	38.71	0.30	0.03	5	6
5	L-lactate dehydrogenase B chain	P07195	36.64	0.35	0.04	4	6
6	Keratin, type II cytoskeletal 75	O95678	59.5	0.40	0.04	5	6
7	Ig lambda chain V-I region NEW	P01701	11.45	0.45	0.04	4	5
8	Keratin, type II cytoskeletal 1b	Q6IFZ6	61.36	0.57	0.05	2	3
9	Neuroblast differentiation-associated protein AHNAK	Q09666	629.1	1.94	0.00	4	6
10	Cathepsin S	P25774	37.5	2.14	0.04	4	3
11	Cation channel sperm-associated protein 3	Q86XQ3	46.42	2.31	0.01	5	5
12	Tubulin-specific chaperone A	O75347	12.86	2.41	0.05	3	4
13	Ribonuclease T2	O00584	29.48	2.42	0.03	5	5
14	Costars family protein	Q9P1F3	9.056	2.92	0.05	5	4
15	Dipeptidyl peptidase 1	P53634	51.85	3.12	0.03	5	4
16	Ig kappa chain V-III region HAH	P18135	14.07	3.34	0.03	5	5
17	Dynein heavy chain 10, axonemal	Q8IVF4	514.8	4.67	0.04	4	5
18	Calcium-activated chloride channel regulator 4	Q14CN2	101.3	5.59	0.04	5	4
19	Proline-rich protein 4	Q16378	15.1	9.60	0.04	4	3

From the 39 statistically significant highly differentiated proteins, 8 had similar trends to those noted in the pooled sample results, including; Glyceraldehyde-3-phosphate dehydrogenase (P04406), S100-A8 (P05109), S100-A9 (P06702), Disulfide-isomerase (P07237), Zinc-alpha-2-glycoprotein (P25311), Cornulin (Q9UBG3), Apolipoprotein A-I (P02647), L-lactate dehydrogenase B chain (P07195).

Interestingly, Zinc-alpha-2-glycoprotein (P25311), showed an increased expression profile in the individual qMS whereas the in the results of the qMS of pooled samples it showed an opposite trend. Another controversial protein was Lipocalin-1 (P31025) in which the individual MS supported the results of the 2DE showing an average increase of more than 3.5-fold in PC patients, but the changes in the MS were not statistically significant.

### Bio-statistical analysis

In order to determine a short panel of discriminative biomarkers, label free qMS results were bio-statistical analyzed utilizing Matlab software R2013a (The MathWorks, Inc, USA). Data was presented in a Volcano plot using the vertical axis (Fig. 2B).

The Biostatistical analysis revealed five highly discriminative proteins; Cytokeratin-14 (P02533), Lactoperoxidase (P22079), Cytokeratin-16 (P08730), Cytokeratin-17 (Q04695) and Peptidyl-prolyl cis–trans isomerase B (P23284).

To further examine the clinical utility of this combination of biomarkers for PC detection, an ROC curve was built. This model yielded a ROC-plot AUC value of 0.910 (95% CI, 0.714 to 1.000; *p* < 0.000001) with 90.0% sensitivity and 90.0% specificity in differentiating PC patients from healthy subjects (Fig. 2C). In other words, 18 out of 20 OF samples showed true positive or true negative results, based on the combined biomarker examination.

## Discussion

Pancreatic cancer (PC) is an aggressive cancer and ranks third in cancer mortality in Israel and 8th worldwide^2,23,24^. Most PC are diagnosed at a late stage demonstrating the need to establish a simpler, non-invasive, cost effective screening tool for PC such as oral fluids (OF).

### Proteomic analysis of pooled OF samples

This is the first study (to our knowledge) characterizing the OF proteome of PC patients. The biomarker candidates identified in our pooled OF samples were compared to previous proteomic studies from other tissues or body fluids. Table 1A summarizes 19 proteins out of 21 with more than threefold changes in expression that were considered as potential biomarkers, details of seven of these proteins are presented below:

i. *Histones* (P62805, P33778, Q96A08) are strongly alkaline proteins which package and organize the DNA into structural units called nucleosomes. Autoantibodies to this protein found in the serum of PC patients have been suggested as potential biomarkers^25,26^.
ii. *Apolipoprotein A-I precursor* has a specific role in lipid metabolism. It is the major component of high-density lipoprotein in plasma and has recently been patented for early diagnosis, screening, therapeutic follow-up and prognosis, as well as diagnosis of relapse of colorectal cancer^27^.
iii. *Myeloperoxidase* is an important factor influencing oxygen dependent mechanisms of pathogen destruction. A significant decrease in the activity of myeloperoxidase has been found in the neutrophils of PC patients^28^.
iv. *Transthyretin precursor* is a serum and cerebrospinal fluid carrier of the thyroid hormone thyroxine (T4) and retinol. Its expression was significantly lower (7.9-fold) in the serum of PC patients^29^.
v. *Lipocalin-1 and Protein S100-A8* were down regulated in PC versus non-neoplastic ductal cells by stable isotope labeling with amino acids in cell culture^30^.
vi. *Transketolase* is up regulated in PC cells compared to healthy pancreatic ducts (3.66-fold increase compared to the 3.18-fold increase we found in OF)^31^.
vii. *Hemopexin* is the highest affinity heme binding protein, protecting the body from the oxidative damage that free heme can cause. This protein has been consistently associated with tumors^30^.

Partial overlap between the two-proteomic screening approaches; 2DE and dimethylation qMS demonstrated the importance of employing different proteomic strategies to maximize identification abilities. The disadvantages of 2DE as a proteomic method including: spots containing more than one protein; limited dynamic range imposed by the gel method; difficulty with hydrophobic proteins; inability to detect proteins with extreme molecular weights and pI values, have been previously described^30^. In order to overcome these limitations, multiple detection methods were used. Furthermore, when a discrepancy was noted between the methods, the label-free qMS on individual samples supported the results of the 2DE upon dimethylation qMS. Nevertheless, the need for extensive individual proteomic analyses and validation is clear.

### Bioinformatic analysis

**Figure 3:**
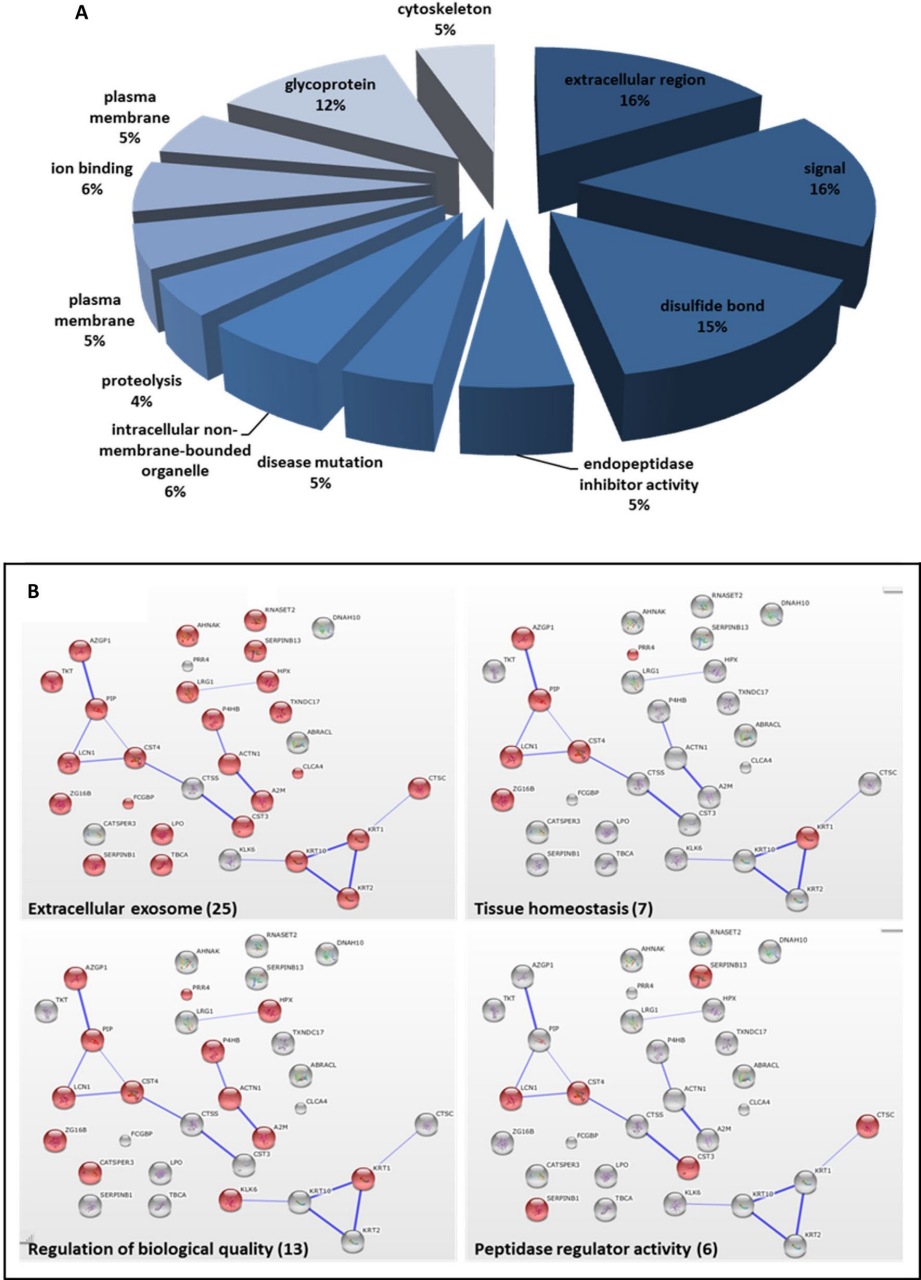
(**A**) David-Kegg Bioinformatics Resources^32^. Classification of proteins with increased expression according to their biological functions. Proteins with more than one biological function were counted multiple times. (**B**) "*String*" online database (http://string-db.org/). Association network of overexpressed proteins in OF of PC patients.

Up and down regulated biomarker candidates were analyzed and clustered according to their molecular and biological functions using David-Kegg Bioinformatics Resources^32^. The expression of 32 proteins increased and 65 had lower levels (> twofold change). The main functional and molecular groups included; signal peptides, glycosylation processes and protease activity (Fig. 3A). These finding are in accordance with extensive bioinformatic analysis of PC biomarker candidates from tumor tissue or patient serum samples^33^. Further analysis utilizing "*String*" bioinformatics website (http://string-db.org/) to explore protein–protein interaction strength revealed four clustered functional groups, including; tissue homeostasis, regulation of biological quality, peptidase regulation activity and extra cellular exosome (Fig. 3B).

In this study 25 out of 32 candidate biomarkers were exosomal proteins. This, most interestingly, is in full agreement with a study by Lau et al*.* discussing the role of tumor-derived exosomes in OF biomarker development^34^. The authors, however, focused on the influence of pancreatic exosomes on OF biomarker development, while the role of the exosomes in the targeted organs remained ambiguous. A partial explanation may be that exosomes not only transport messenger molecules from the pancreas to the salivary glands, but also deliver biomarkers to OF. Whether these are the original pancreatic exosomes or newly secreted vesicles from the salivary glands, should be examined further.

Similarly, an in vitro examination showed that breast cancer derived exosomes interact with the salivary glands and alter the composition of salivary gland cell-derived exosome-like macrovesicles in the transcriptome and proteome^35^.

Because a solitary biomarker is unlikely to detect a particular cancer with high specificity and sensitivity, we evaluated combinations of the identified biomarkers using an ROC analysis. We calculated high ROC AUC values indicating that the predictive utility increased substantially, enabling the identification of a group of five biomarker candidates. Three Cytokeratin types (14, 16 and 17), involved in the regulation of cellular properties and functions, including apico-basal polarization, motility, cell size, protein synthesis and membrane traffic and signaling were selected. In many cases, their presence or absence has prognostic significance for cancer patients^36^. The role of cytokeratins in pancreatic cancer and the ability to utilize them as biomarkers is widely discussed in the literature^37,38^. For example Keratin 17 was proven to be a novel negative prognostic biomarker for pancreatic cancer^39^.

The remaining two proteins with elevated levels in OF of PC patients and included in our biomarker combination were Lactoperoxidase and Peptidyl-prolyl cis–trans isomerase B. The latter is also called Cyclophilin B (CypB) and is a 21-kDa protein belonging to the cyclophilin family of peptidyl-prolyl cis–trans isomerase. It promotes alterations in protein conformation and influences cell growth, proliferation, and motility^40^.

Enhanced expression of CypB in malignant breast epithelium may contribute to the pathogenesis of the disease^41^. Moreover, elevated levels of CypB have been found in sera of PC patients and this protein has been suggested as a serum biomarker for PC^42^.

The comparison of pooled sample results to individual qMS analysis showed partial overlap. Approximately 33% of the proteins with the highest expression fold change and lowest p-value identified in the individual samples presented similar expression trends in pooled samples.

Furthermore, CypB, one of the five discriminative biomarkers found in the individual qMS analysis, was related to the down regulation of two S100 proteins. Both the pooled and individual qMS analysis showed decreased expression levels in these proteins. It was previously claimed that pooling serum samples may cause a ~ 50% loss of potential biomarkers^43^. The results of the current study support this argument; yet also show the advantages of the pooling strategy as an initial step before performing extensive examinations on individual samples. Pooled sample analysis enabled a relatively low-cost and rapid "proof of concept" examination. Clearly, validation using individual samples is required to understand the diagnostic potential of the biomarker combination.

## Concluding remarks

Enhanced proteomic characterization of the oral fluids of PC patients revealed a profile of differentially expressed proteins. Bioinformatic analysis of OF was in accordance with previous studies of proteins expressed in PC in tissues, pancreatic juice or serum. Moreover, an extensive label free qMS analysis revealed a group of proteins, which may be used as a highly specific, and sensitive OF based test for PC test. A larger study is required for A. Exploring the accuracy of the combined 5 biomarkers that were found in this study, utilizing different proteomic technology (e.g. Elisa, Western blot, lateral flow immunoassay etc.).

B. validation and identifying high-risk groups in order to enable an early diagnosis, screening, therapeutic follow-up and prognosis and diagnosis of relapse in relation to PC using OF.

## Supplementary Information

Supplementary Datasets.

## Abbreviations

OF: Oral fluids
PC: Pancreatic cancer
