# Supplementary material for: Possible proteomic biomarkers for the detection of pancreatic cancer in oral fluids
Source: Sci Rep. 2020 Dec 15;10:21995. doi: 10.1038/s41598-020-78922-x (PMC7738525; doi:10.1038/s41598-020-78922-x)
Supplement: Supplementary file 1 — Supplementary Datasets. [file 41598_2020_78922_MOESM1_ESM.docx]

# Possible Proteomic Biomarkers for the Detection of Pancreatic Cancer in Oral Fluids

[Deutsch](mailto:omerde@ekmd.huji.ac.il) O.^1^*, Haviv Y.^2^*, Krief G.^1^, Keshet N.^2^, Westreich R.^3^, Stemmer S^4^, Zaks B.^1^, Navat S.^1^, Lahav O. ^1^, Aframian D.J.^2^*, Palmon A.^1^*

# Supplementary data A

Drugs conceived by PC patients and xerostomia severity

| *Xerostomia severity** | *Number of subjects conceiving drug* | *Generic name* | *Commercial name* |
| --- | --- | --- | --- |
| 1 | 2 | Omeprazole | Losec |
| 1 | 2 | Ramipril | Tritace |
| 1 | 2 | Hydrochlorothiazide | Disothiazide |
| 1 | 1 | calcium carbonate | Caltrate |
| 1 | 2 | Amlodipine as maleate | Amlow |
| 0 | 1 | Alendronic acid | Fosalan |
| 1 | 2 | Carvedilol | Dimiton |
| 1 | 4 | Atenolol | Normiten |
| 3 | 1 | Furosemide | Fusid |
| 1 | 1 | Dutasteride | Avodart |
| 1 | 2 | Simvastatin | Simvacor |
| 0 | 2 | Insulin | Insulin |
| 1 | 1 | Atenolol | Normalol |
| 2 | 1 | Enalapril Maletate | Enaladex |
| 1 | 3 | Acetylsalicylic acid | aspirin |
| 1 | 2 | Dipyrone | Optalgin |
| 2 | 1 | Escitalopram | Cypralex |
| 1 | 1 | Sotalol | Betapace |
| 1 | 1 | Atorvastatin calcium | Lipitor |
| 1 | 2 | Tamsulosin hydrochloride | Omnic |
| 0 | 2 | Metformin hydrochloride | Glucophage |
| 1 | 1 | Rosuvastatin | Crestor |
| 1 | 2 | Repaglinide | Novonorm |
| 1 | 2 | Simvastatin | Simovil |
| 2 | 1 | fluticasone propionate and salmeterol xinafoate | Fluticasone |
| 1 | 1 | Montelukast | Singulair |
| 1 | 1 | Valsartan | Diovan |
| 3 | 1 | Amitriptyline Hydrochloride | Elatrol |
| 1 | 1 | Famotidine | Famotidine |
| 1 | 2 | Levothyroxine Sodium | Eltroxin |
| 1 | 1 | Acetylsalicylic acid | Cartia |
| 1 | 1 | Verapamil hydrochloride | Ikapress |

Drugs conceived by healthy volunteers and xerostomia severity

| *Xerostomia severity** | *Number of subjects conceiving drug* | *Generic name* | *Commercial name* |
| --- | --- | --- | --- |
| 1 | 1 | Acetylsalicylic acid | Micropirin |
| 1 | 2 | Simvastatin | Simvastatin |
| 1 | 1 | Atorvastatin calcium | Lipitor |
| 1 | 1 | Amoxicillin trihydrate | Moxypen |
| 1 | 3 | Acetylsalicylic acid | Aspirin |

* ***Xerostomia severity*** indicates the percentage of drug consumers suffer from dry mouth ([www.drymouth.info](http://www.drymouth.info)):

1. No evidence for xerostomia symptoms
2. Xerostomia in less than 1% of consumers
3. Xerostomia in 1-10% of consumers
4. Xerostomia in more than 10% of consumers

# Supplementary data B

A list of proteins identified by dimethylation followed by LC-MS/MS of pooled PC and healthy OF samples and expression profile ratio.

| Serial  No. | | | | Protein Identification | Accession No. | MW (Da) | Peptides No. | PC/  Healthy |
| --- | --- | --- | --- | --- | --- | --- | --- | --- |
| 1 | | Histone H4 | | | P62805 | 11360 | 3 | 0.02 |
| 2 | | Histone H2B type 1-B | | | P33778 | 13942 | 2 | 0.03 |
| 3 | | 6-phosphogluconate dehydrogenase, decarboxylating | | | P52209 | 53106 | 2 | 0.04 |
| 4 | | Basic salivary proline-rich protein 2 precursor | | | P02812 | 40775 | 2 | 0.05 |
| 5 | | Histone H2B type 1-A | | | Q96A08 | 14159 | 2 | 0.06 |
| 6 | | Azurocidin precursor | | | P20160 | 26869 | 3 | 0.07 |
| 7 | | Apolipoprotein A-I precursor | | | P02647 | 30759 | 7 | 0.08 |
| 8 | | Alpha-amylase 1 precursor | | | P04745 | 57731 | 31 | 0.16 |
| 9 | | Myeloperoxidase precursor | | | P05164 | 83815 | 9 | 0.16 |
| 10 | | Protein S100-A8 | | | P05109 | 10828 | 6 | 0.19 |
| 11 | | Transthyretin precursor | | | P02766 | 15877 | 6 | 0.22 |
| 12 | | Lipocalin-1 precursor | | | P31025 | 19238 | 12 | 0.23 |
| 13 | | Protein S100-A9 | | | P06702 | 13234 | 7 | 0.24 |
| 14 | | Short palate, lung and nasal epithelium carcinoma-associated protein 2 precursor | | | Q96DR5 | 26995 | 6 | 0.24 |
| 15 | | Hemoglobin subunit alpha | | | P69905 | 15248 | 9 | 0.25 |
| 16 | | Small proline-rich protein 2A | | | P35326 | 7960 | 3 | 0.25 |
| 17 | | Hemoglobin subunit delta | | | P02042 | 16045 | 2 | 0.26 |
| 18 | | Fibrinogen alpha chain precursor [Contains: Fibrinopeptide A] | | | P02671 | 94914 | 5 | 0.31 |
| 19 | | Serum albumin precursor | | | P02768 | 69322 | 14 | 0.31 |
| 20 | | Hemoglobin subunit beta | | | P68871 | 15988 | 20 | 0.33 |
| 21 | | Vitamin D-binding protein precursor | | | P02774 | 52929 | 5 | 0.34 |
| 22 | | Complement C3 precursor [Contains: Complement C3 beta chain; Complement C3 alpha chain…] | | | P01024 | 187029 | 27 | 0.35 |
| 23 | | Alpha-1B-glycoprotein precursor | | | P04217 | 54239 | 5 | 0.35 |
| 24 | | Alpha-1-acid glycoprotein 1 precursor | | | P02763 | 23497 | 8 | 0.37 |
| 25 | | Actin, cytoplasmic 1 | | | P60709 | 41710 | 12 | 0.38 |
| 26 | | L-lactate dehydrogenase B chain | | | P07195 | 36615 | 2 | 0.39 |
| 27 | | Leukotriene A-4 hydrolase | | | P09960 | 69241 | 2 | 0.40 |
| 28 | | Fibrinogen gamma chain precursor | | | P02679 | 51479 | 6 | 0.40 |
| 29 | | Involucrin | | | P07476 | 68427 | 2 | 0.44 |
| 30 | | Metalloproteinase inhibitor 1 precursor | | | P01033 | 23156 | 2 | 0.46 |
| 31 | | Glyceraldehyde-3-phosphate dehydrogenase | | | P04406 | 36030 | 8 | 0.48 |
| 32 | | Fibrinogen beta chain precursor [Contains: Fibrinopeptide B] | | | P02675 | 55892 | 5 | 0.49 |
| 33 | | Protein-glutamine gamma-glutamyltransferase E precursor | | | Q08188 | 76584 | 6 | 0.49 |
| 34 | | Beta-2-glycoprotein 1 precursor - Homo sapiens (Human) | | | P02749 | 38273 | 3 | 0.49 |
| 35 | | Keratin, type I cytoskeletal 13 | | | P13646 | 49555 | 3 | 0.49 |
| 36 | | Ig alpha-1 chain C region | | | P01876 | 37631 | 27 | 0.54 |
| 37 | | Serotransferrin precursor | | | P02787 | 77000 | 56 | 0.54 |
| 38 | | Vimentin | | | P08670 | 53619 | 5 | 0.54 |
| 39 | | Alpha-1-acid glycoprotein 2 precursor | | | P19652 | 23588 | 2 | 0.54 |
| 40 | | Desmoglein-1 precursor | | | Q02413 | 113644 | 3 | 0.57 |
| 41 | | Ig kappa chain C region | | | P01834 | 11602 | 14 | 0.58 |
| 42 | | Zinc-alpha-2-glycoprotein precursor | | | P25311 | 33851 | 32 | 0.58 |
| 43 | | Cornulin | | | Q9UBG3 | 53502 | 4 | 0.58 |
| 44 | | Phosphoglycerate mutase 1 | | | P18669 | 28786 | 2 | 0.58 |
| 45 | | Ig gamma-1 chain C region | | | P01857 | 36083 | 11 | 0.58 |
| 46 | | Ig heavy chain V-III region VH26 precursor | | | P01764 | 12574 | 6 | 0.58 |
| 47 | | Complement factor B precursor | | | P00751 | 85479 | 2 | 0.59 |
| 48 | | Aldehyde dehydrogenase, dimeric NADP-preferring | | | P30838 | 50347 | 3 | 0.60 |
| 49 | | Glucose-6-phosphate isomerase | | P06744 | 63107 | 11 | 0.63 |  |
| 50 | | Lactotransferrin precursor | | P02788 | 78132 | 65 | 0.64 |  |
| 51 | | Cystatin-D precursor | | P28325 | 16070 | 9 | 0.64 |  |
| 52 | | | Mucin-5AC precursor | | P98088 | 526278 | 6 | 0.64 |
| 53 | | | Ceruloplasmin precursor | | P00450 | 122128 | 12 | 0.65 |
| 54 | | | Uncharacterized protein C6orf58 precursor | | Q6P5S2 | 37902 | 18 | 0.66 |
| 55 | | | Deleted in malignant brain tumors 1 protein precursor | | Q9UGM3 | 260567 | 14 | 0.67 |
| 56 | | | Carbonic anhydrase 6 precursor | | P23280 | 35345 | 15 | 0.67 |
| 57 | | | Pyruvate kinase isozymes M1/M2 | | P14618 | 57900 | 8 | 0.67 |
| 58 | | | Prolactin-inducible protein homolog precursor | | A0A885 | 16493 | 4 | 0.67 |
| 59 | | | Coronin-1A | | P31146 | 50994 | 4 | 0.67 |
| 60 | | | Matrix metalloproteinase-9 precursor | | P14780 | 78377 | 14 | 0.68 |
| 61 | | | Haptoglobin precursor [Contains: Haptoglobin alpha chain; Haptoglobin beta chain] | | P00738 | 45177 | 17 | 0.69 |
| 62 | | | L-lactate dehydrogenase A chain | | P00338 | 36665 | 8 | 0.69 |
| 63 | | | Alpha-actinin-4 | | O43707 | 104789 | 2 | 0.70 |
| 64 | | | Polymeric-immunoglobulin receptor precursor | | P01833 | 83232 | 47 | 0.73 |
| 65 | | | Mucin-5B precursor | | Q9HC84 | 590135 | 89 | 0.73 |
| 66 | | | Cystatin-SN precursor | | P01037 | 16351 | 13 | 0.74 |
| 67 | | | Desmocollin-2 precursor | | Q02487 | 99899 | 4 | 0.74 |
| 68 | | | Bactericidal/permeability-increasing protein-like 1 precursor | | Q8N4F0 | 49142 | 5 | 0.75 |
| 69 | | | Haptoglobin-related protein precursor | | P00739 | 38983 | 14 | 0.75 |
| 70 | | | Cystatin-A | | P01040 | 11000 | 7 | 0.76 |
| 71 | | | 14-3-3 protein zeta/delta | | P63104 | 27728 | 11 | 0.78 |
| 72 | | | Ig gamma-2 chain C region | | P01859 | 35862 | 4 | 0.79 |
| 73 | | | Fatty acid-binding protein, epidermal | | Q01469 | 15155 | 9 | 0.79 |
| 74 | | | Cystatin-SA precursor | | P09228 | 16434 | 6 | 0.81 |
| 75 | | | Cystatin-B | | P04080 | 11133 | 7 | 0.83 |
| 76 | | | Proactivator polypeptide precursor [Contains: Saposin-A] | | P07602 | 58074 | 9 | 0.84 |
| 77 | | | Thioredoxin | | P10599 | 11730 | 6 | 0.84 |
| 78 | | | Galectin-3-binding protein precursor | | Q08380 | 65289 | 9 | 0.84 |
| 79 | | | Lactoperoxidase precursor | | P22079 | 80237 | 14 | 0.85 |
| 80 | | | Serine protease inhibitor Kazal-type 5 precursor | | Q9NQ38 | 120680 | 4 | 0.85 |
| 81 | | | Ig kappa chain V-IV region Len | | P01625 | 12632 | 2 | 0.86 |
| 82 | | | Epididymal secretory protein E1 precursor | | P61916 | 16559 | 2 | 0.86 |
| 83 | | | Phosphoglycerate kinase 1 | | P00558 | 44586 | 3 | 0.88 |
| 84 | | | Desmoglein-3 precursor | | P32926 | 107436 | 11 | 0.88 |
| 85 | | | Calmodulin-like protein 3 | | P27482 | 16880 | 11 | 0.88 |
| 86 | | | Beta-2-microglobulin precursor [Contains: Beta-2-microglobulin form pI 5.3] | | P61769 | 13706 | 6 | 0.89 |
| 87 | | | Adenylyl cyclase-associated protein 1 | | Q01518 | 51823 | 12 | 0.89 |
| 88 | | | Ig mu chain C region | | P01871 | 49526 | 9 | 0.91 |
| 89 | | | Cysteine-rich secretory protein 3 precursor | | P54108 | 27612 | 4 | 0.92 |
| 90 | | | Protein S100-A11 | | P31949 | 11733 | 6 | 0.93 |
| 91 | | | Calmodulin-like protein 5 | | Q9NZT1 | 15911 | 3 | 0.95 |
| 92 | | | Myeloblastin precursor | | P24158 | 27789 | 3 | 0.96 |
| 93 | | | F-actin-capping protein subunit beta | | P47756 | 31331 | 2 | 0.98 |
| 94 | | | CD59 glycoprotein precursor | | P13987 | 14168 | 2 | 0.99 |
| 95 | | | Acyl-CoA-binding protein | | P07108 | 10038 | 5 | 1.05 |
| 96 | | | Ly6/PLAUR domain-containing protein 3 precursor | | O95274 | 35948 | 3 | 1.05 |
| 97 | | | Alpha-enolase, lung specific | | Q05524 | 49446 | 8 | 1.07 |
| 98 | | | Ig lambda chain V-III region SH | | P01714 | 11386 | 2 | 1.09 |
| 99 | | | Triosephosphate isomerase | | P60174 | 26653 | 20 | 1.10 |
| 100 | | | Trefoil factor 3 precursor | | Q07654 | 8635 | 3 | 1.10 |
| 101 | | | Plastin-1 | | Q14651 | 70308 | 2 | 1.10 |
| 102 | | | Cystatin-C precursor | | P01034 | 15789 | 4 | 1.11 |
| 103 | | | Ubiquitin | | P62988 | 8560 | 6 | 1.12 |
| 104 | | | Carcinoembryonic antigen-related cell adhesion molecule 5 precursor | | P06731 | 76748 | 2 | 1.13 |
| 105 | | | Interleukin-1 receptor antagonist protein precursor | | P18510 | 20042 | 4 | 1.14 |
| 106 | | | SH3 domain-binding glutamic acid-rich-like protein 3 - | | Q9H299 | 10431 | 4 | 1.14 |
| 107 | | | Beta-enolase | | P13929 | 46957 | 2 | 1.16 |
| 108 | | | Heat shock cognate 71 kDa protein | | P11142 | 70854 | 8 | 1.17 |
| 109 | | | Neutrophil gelatinase-associated lipocalin precursor | | P80188 | 22574 | 13 | 1.18 |
| 110 | | | Peroxiredoxin-1 | | Q06830 | 22096 | 9 | 1.18 |
| 111 | | | Phosphatidylethanolamine-binding protein 1 | | P30086 | 21044 | 4 | 1.20 |
| 112 | | | Protein UNQ773/PRO1567 precursor | | Q96DA0 | 22725 | 6 | 1.20 |
| 113 | | | Gelsolin precursor | | P06396 | 85644 | 14 | 1.21 |
| 114 | | | Cytochrome c | | P99999 | 11741 | 2 | 1.21 |
| 115 | | | SH3 domain-binding glutamic acid-rich-like protein | | O75368 | 12766 | 2 | 1.22 |
| 116 | | | Leucine-rich alpha-2-glycoprotein precursor | | P02750 | 38154 | 5 | 1.23 |
| 117 | | | Glutathione S-transferase P | | P09211 | 23341 | 12 | 1.24 |
| 118 | | | Peptidoglycan recognition protein precursor | | O75594 | 21717 | 4 | 1.24 |
| 119 | | | Fructose-bisphosphate aldolase A | | P04075 | 39395 | 7 | 1.24 |
| 120 | | | Macrophage migration inhibitory factor | | P14174 | 12468 | 2 | 1.24 |
| 121 | | | Serpin B3 | | P29508 | 44537 | 10 | 1.25 |
| 122 | | | Transaldolase | | P37837 | 37516 | 7 | 1.26 |
| 123 | | | Alpha-enolase | | P06733 | 47139 | 18 | 1.27 |
| 124 | | | Ig kappa chain V-I region EU | | P01598 | 11781 | 2 | 1.28 |
| 125 | | | Heat shock 70 kDa protein homolog | | P08418 | 69832 | 9 | 1.29 |
| 126 | | | Coactosin-like protein | | Q14019 | 15935 | 2 | 1.30 |
| 127 | | | Actin, aortic smooth muscle | | P62736 | 41982 | 17 | 1.32 |
| 128 | | | Heat shock 70 kDa protein 1L | | P34931 | 70331 | 10 | 1.32 |
| 129 | | | Ig heavy chain V-I region HG3 precursor | | P01743 | 12937 | 2 | 1.32 |
| 130 | | | Transgelin-2 | | P37802 | 22377 | 7 | 1.33 |
| 131 | | | 14-3-3 protein sigma | | P31947 | 27757 | 9 | 1.34 |
| 132 | | | Ig kappa chain V-I region DEE | | P01597 | 11654 | 4 | 1.34 |
| 133 | | | Ig mu heavy chain disease protein | | P04220 | 43030 | 14 | 1.34 |
| 134 | | | 78 kDa glucose-regulated protein homolog precursor | | Q6BZH1 | 74539 | 2 | 1.34 |
| 135 | | | Submaxillary gland androgen-regulated protein 3 homolog B precursor | | P02814 | 8182 | 2 | 1.35 |
| 136 | | | Ig kappa chain V-I region AG | | P01593 | 11985 | 5 | 1.35 |
| 137 | | | Ig heavy chain V-III region BRO | | P01766 | 13218 | 2 | 1.36 |
| 138 | | | Ig heavy chain V-III region TIL | | P01765 | 12348 | 2 | 1.36 |
| 139 | | | Alpha-1-antitrypsin precursor | | P01009 | 46707 | 13 | 1.37 |
| 140 | | | Cornifin-A | | P35321 | 9876 | 4 | 1.37 |
| 141 | | | Ig kappa chain V-III region VG precursor | | P04433 | 12567 | 2 | 1.38 |
| 142 | | | Ig alpha-2 chain C region | | P01877 | 36485 | 8 | 1.39 |
| 143 | | | Clusterin precursor | | P10909 | 52461 | 4 | 1.39 |
| 144 | | | Protein disulfide-isomerase precursor | | P07237 | 57081 | 17 | 1.40 |
| 145 | | | Kallikrein-1 precursor | | P06870 | 28871 | 7 | 1.40 |
| 146 | | | Ig lambda chain V-III region LOI | | P80748 | 11928 | 4 | 1.40 |
| 147 | | | Protein S100-A7 | | P31151 | 11450 | 8 | 1.40 |
| 148 | | | Rho GDP-dissociation inhibitor 2 | | P52566 | 22974 | 6 | 1.41 |
| 149 | | | Catalase | | P04040 | 59719 | 16 | 1.42 |
| 150 | | | Heat shock 70 kDa protein 1 | | P08107 | 70009 | 8 | 1.42 |
| 151 | | | Immunoglobulin J chain | | P01591 | 15585 | 9 | 1.44 |
| 152 | | | WAP four-disulfide core domain protein 2 precursor | | Q14508 | 12984 | 4 | 1.45 |
| 153 | | | Epithelial cadherin precursor | | P12830 | 97396 | 3 | 1.47 |
| 154 | | | Ig kappa chain V-I region CAR | | P01596 | 11696 | 3 | 1.50 |
| 155 | | | Ig kappa chain V-II region Cum | | P01614 | 12668 | 2 | 1.50 |
| 156 | | | Ig lambda chain C regions | | P01842 | 11230 | 10 | 1.51 |
| 157 | | | 78 kDa glucose-regulated protein precursor | | P11021 | 72289 | 12 | 1.51 |
| 158 | | | Ig heavy chain V-II region ARH-77 precursor | | P06331 | 16218 | 2 | 1.51 |
| 159 | | | Transcobalamin-1 precursor | | P20061 | 48176 | 12 | 1.54 |
| 160 | | | Ig heavy chain V-III region WEA | | P01763 | 12249 | 2 | 1.55 |
| 161 | | | Profilin-1 | | P07737 | 15045 | 16 | 1.56 |
| 162 | | | Peptidyl-prolyl cis-trans isomerase A | | P62937 | 18001 | 8 | 1.57 |
| 163 | | | Plastin-2 | | P13796 | 70245 | 17 | 1.59 |
| 164 | | | Nucleobindin-2 precursor | | P80303 | 50191 | 7 | 1.59 |
| 165 | | | Cofilin-1 | | P23528 | 18491 | 2 | 1.61 |
| 166 | | | Ribonuclease T2 precursor | | O00584 | 29462 | 2 | 1.62 |
| 167 | | | Ig heavy chain V-III region GAL | | P01781 | 12722 | 6 | 1.66 |
| 168 | | | Leukocyte elastase inhibitor | | P30740 | 42715 | 5 | 1.68 |
| 169 | | | Small proline-rich protein 3 | | Q9UBC9 | 18142 | 5 | 1.70 |
| 170 | | | IgGFc-binding protein precursor | | Q9Y6R7 | 571692 | 9 | 1.74 |
| 171 | | | Calmodulin | | P62158 | 16827 | 3 | 1.75 |
| 172 | | | Cornifin-B | | P22528 | 9881 | 2 | 1.78 |
| 173 | | | | Cystatin-S precursor | P01036 | 16204 | 10 | 1.91 |
| 174 | | | | Ig kappa chain V-III region SIE | P01620 | 11768 | 6 | 1.94 |
| 175 | | | | Keratin, type II cytoskeletal 1 | P04264 | 65978 | 4 | 2.15 |
| 176 | | | | Keratin, type II cytoskeletal 2 epidermal | P35908 | 65825 | 4 | 2.29 |
| 177 | | | | Alpha-actinin-1 | P12814 | 102993 | 4 | 2.49 |
| 178 | | | | Prolactin-inducible protein precursor | P12273 | 16562 | 10 | 2.59 |
| 179 | | | | Transketolase | P29401 | 67835 | 11 | 3.18 |
| 180 | | | | Keratin, type I cytoskeletal 10 | P13645 | 59475 | 2 | 4.57 |
| 181 | | | | Hemopexin precursor | P02790 | 51643 | 13 | 4.99 |
| 182 | | | | Alpha-2-macroglobulin precursor | P01023 | 163174 | 41 | 8.06 |

# Supplementary data C

Differentially expressed proteins identiﬁed by 2-DE and MS analysis of pooled PC and control (C) oral fluid samples.

(+) over-expression and (-) under-expression in PC samples.

| PC vs C | Sequence Coverage (%) | Matched Peptides | theor. MW | Accesion no. | Protein Identification | Spot |
| --- | --- | --- | --- | --- | --- | --- |
| + | 13.4 | 9 | 57081 | P07237 | Protein disulfide-isomerase | 1 |
|  | 1.3 | 4 | 571641 | Q9Y6R7 | IgGFc-binding protein |  |
| + | 7.4 | 2 | 34237 | P25311 | Zinc-alpha-2-glycoprotein | 2 |
| + | 13.1 | 3 | 34237 | P25311 | Zinc-alpha-2-glycoprotein | 3 |
|  | 5.4 | 2 | 44248 | Q9UIV8 | Serpin B13 |  |
| + | 5.3 | 2 | 42715 | P30740 | Leukocyte elastase inhibitor | 4 |
| + | 10.6 | 4 | 42715 | P30740 | Leukocyte elastase inhibitor | 5 |
| - | 40.0 | 5 | 15155 | Q01469 | Fatty acid-binding protein, epidermal | 6 |
| - | 21.4 | 3 | 11133 | P04080 | Cystatin-B | 7 |
|  | 17.0 | 2 | 15988 | P68871 | Hemoglobin subunit beta |  |
|  | 3.5 | 2 | 31203 | Q9Y275 | Tumor necrosis factor ligand superfamily member 13B |  |
| - | 21.4 | 3 | 11133 | P04080 | Cystatin-B | 8 |
|  | 17.0 | 2 | 15988 | P68871 | Hemoglobin subunit beta |  |
| - | 16.8 | 2 | 13706 | P61769 | Beta-2-microglobulin | 9 |
| - | 11.3 | 7 | 83232 | P01833 | Polymeric immunoglobulin receptor | 10 |
|  | 6.0 | 3 | 49276 | P01871 | Ig mu chain C region |  |
|  | 5.1 | 2 | 37631 | P01876 | Ig alpha-1 chain C region |  |
| - | 12.4 | 7 | 69241 | P09960 | Leukotriene A-4 hydrolase | 11 |
|  | 16.1 | 5 | 37631 | P01876 | Ig alpha-1 chain C region |  |
|  | 6.9 | 4 | 69322 | P02768 | Serum albumin |  |
|  | 65.1 | 4 | 11602 | P01834 | Ig kappa chain C region | 12 |
| - | 16.7 | 2 | 11654 | P01597 | Ig kappa chain V-I region DEE |  |
|  | 24.8 | 2 | 11768 | P01620 | Ig kappa chain V-III region SIE |  |
|  | 16.7 | 2 | 11985 | P01593 | Ig kappa chain V-I region AG |  |
| + | 23.3 | 4 | 19238 | P31025 | Lipocalin-1 | 13 |
| + | 23.3 | 4 | 19238 | P31025 | Lipocalin-1 | 14 |
| + | 23.3 | 4 | 19238 | P31025 | Lipocalin-1 | 15 |
